# Comparative effectiveness and safety of DOACs vs. VKAs in treatment of left ventricular thrombus- a meta-analysis update

**DOI:** 10.1186/s12959-024-00585-9

**Published:** 2024-03-01

**Authors:** Tong Hu, Changli Chen, Kellina Maduray, Wenqiang Han, Tongshuai Chen, Jingquan Zhong

**Affiliations:** 1 National Key Laboratory for Innovation and Transformation of Luobing Theory; The Key Laboratory of Cardiovascular Remodeling and Function Research, Chinese Ministry of Education, Chinese National Health Commission and Chinese Academy of Medical Sciences; Department of Cardiology, Qilu Hospital of Shandong University, 107 Wen Hua Xi Road, Jinan, 250012 China; 2 Department of Cardiology, Qilu Hospital (Qingdao), Cheeloo College of Medicine, Shandong University, 758 Hefei Road, Qingdao, 266035 Shandong China

## Abstract

**Background and objective:**

Left ventricular thrombus (LVT) formation in patients with acute myocardial infarction (AMI) or cardiomyopathies is not uncommon. The optimal oral anticoagulation therapy for resolving LVT has been under intense debate. Vitamin K antagonists (VKAs) remain the anticoagulant of choice for this condition, according to practice guidelines. Evidence supporting the use of direct oral anticoagulants (DOACs) in the management of LVT continues to grow. We performed a systematic review and meta-analysis to compare the efficacy and safety of DOACs versus VKAs.

**Methods:**

A comprehensive literature search was carried out in PubMed, Cochrane Library, Web of Science, Embase, and Scopus databases in July 2023. The efficacy outcomes of this study were thrombus resolution, ischemic stroke, systemic embolism, stroke/systemic embolism, all-cause mortality, and adverse cardiovascular events. The safety outcomes were any bleeding, major bleeding, and intracranial hemorrhage. A total of twenty-seven eligible studies were included in the meta-analysis. Data were analyzed utilizing Stata software version 15.1.

**Results:**

There was no significant difference between DOACs and VKAs with regard to LVT resolution (RR = 1.00, 95% CI 0.95–1.05, *P* = 0.99). In the overall analysis, DOACs significantly reduced the risk of stroke (RR = 0.74, 95% CI 0.57–0.96, *P* = 0.021), all-cause mortality (RR = 0.70, 95% CI 0.57–0.86, *P* = 0.001), any bleeding (RR = 0.75, 95% CI 0.61–0.92, *P* = 0.006) and major bleeding (RR = 0.67, 95% CI 0.52–0.85, *P* = 0.001) when compared to VKAs. Meanwhile, in the sub-analysis examining randomized controlled trials (RCTs), the aforementioned outcomes no longer differed significantly between the DOACs and VKAs groups. The incidences of systemic embolism (RR = 0.81, 95% CI 0.54–1.22, *P* = 0.32), stroke/systemic embolism (RR = 0.85, 95% CI 0.72–1.00, *P* = 0.056), intracranial hemorrhage (RR = 0.59, 95% CI 0.23–1.54, *P* = 0.28), and adverse cardiovascular events (RR = 0.99, 95% CI 0.63–1.56, *P* = 0.92) were comparable between the DOACs and VKAs groups. A subgroup analysis showed that patients treated with rivaroxaban had a significantly lower risk of stroke (RR = 0.24, 95% CI 0.08–0.72, *P* = 0.011) than those in the VKAs group.

**Conclusion:**

With non-inferior efficacy and superior safety, DOACs are promising therapeutic alternatives to VKAs in the treatment of LVT. Further robust investigations are warranted to confirm our findings.

**Supplementary Information:**

The online version contains supplementary material available at 10.1186/s12959-024-00585-9.

## Introduction

Left ventricular thrombus (LVT) is a complication of acute myocardial infarction (AMI) or nonischemic cardiomyopathy, leading to an increased risk of ischemic stroke and systemic embolism [1, 2]. Although the incidence of LVT after AMI has declined over the past decades, owing to the widely promoted early revascularization therapies, 4–39% of patients develop LVT after anterior ST-segment elevation myocardial infarction (STEMI) [3]. LVT is detected in up to 36% of patients with dilated cardiomyopathy (DCM) [4].

Vitamin K antagonists (VKAs), primarily warfarin, were recommended by the 2013 ACCF/AHA Guideline for the Management of STEMI for the treatment of LVT [5]. Previous randomized controlled trials (RCTs) have demonstrated that direct oral anticoagulants (DOACs) can be effectively and safely used in patients with atrial fibrillation and venous thrombosis [6, 7]. Compared to conventional VKAs, DOACs have several superiorities, including lower risk of bleeding, convenience of use, and fewer interactions with diet or drugs.

The most recent statement from the American Heart Association (AHA) recommends DOACs as an appropriate alternative to traditional VKAs for the resolution of LVT [1]. However, whether DOACs can become the preferred option remains unclear.

Given the consistently growing evidence supporting the use of DOACs for patients with LVT [8, 9], there is an urgent need to update a systematic review and meta-analysis. This study aims to compare the efficacy and safety of DOACs versus VKAs in the setting of LVT.

## Method

This systemic review and meta-analysis was conducted in accordance with PRISMA (Preferred Reporting Items for Systematic Reviews and Meta-Analyses) guidelines [10]. The protocol of this review was registered in PROSPERO (registration number: CRD42023444725).

### Electronic searches

We thoroughly searched PubMed, Cochrane Library, Web of Science, Embase, and Scopus databases for eligible studies published before July 2023. The retrieval terms of this study are as follows. (“left ventricular thrombus” OR “left ventricular thrombi”) AND (“anticoagulation” OR “warfarin” OR “vitamin K antagonist” OR “non-vitamin K antagonist” OR “direct oral anticoagulant” OR “novel oral anticoagulant” OR “rivaroxaban” OR “apixaban” OR “edoxaban” OR “dabigatran”). The electronic search results were imported into EndNote X9. The titles and abstracts were screened, and the full text of potentially relevant studies was reviewed. We also manually searched the reference lists of the included studies to identify additional eligible articles. The detailed search strategies of databases are provided in Supplemental Table 1.

### Outcomes

The efficacy outcomes of this study were thrombus resolution, ischemic stroke, systemic embolism, a composite of stroke and systemic embolism, all-cause mortality, and adverse cardiovascular events (cardiovascular death, AMI and cardiovascular hospitalization). Thrombus resolution was assessed by transthoracic echocardiography (TTE), transesophageal echocardiography (TEE) or cardiac magnetic resonance imaging (CMR). The safety outcomes were any bleeding, major bleeding, and intracranial hemorrhage.

### Eligibility criteria and study selection

**Fig. 1:**
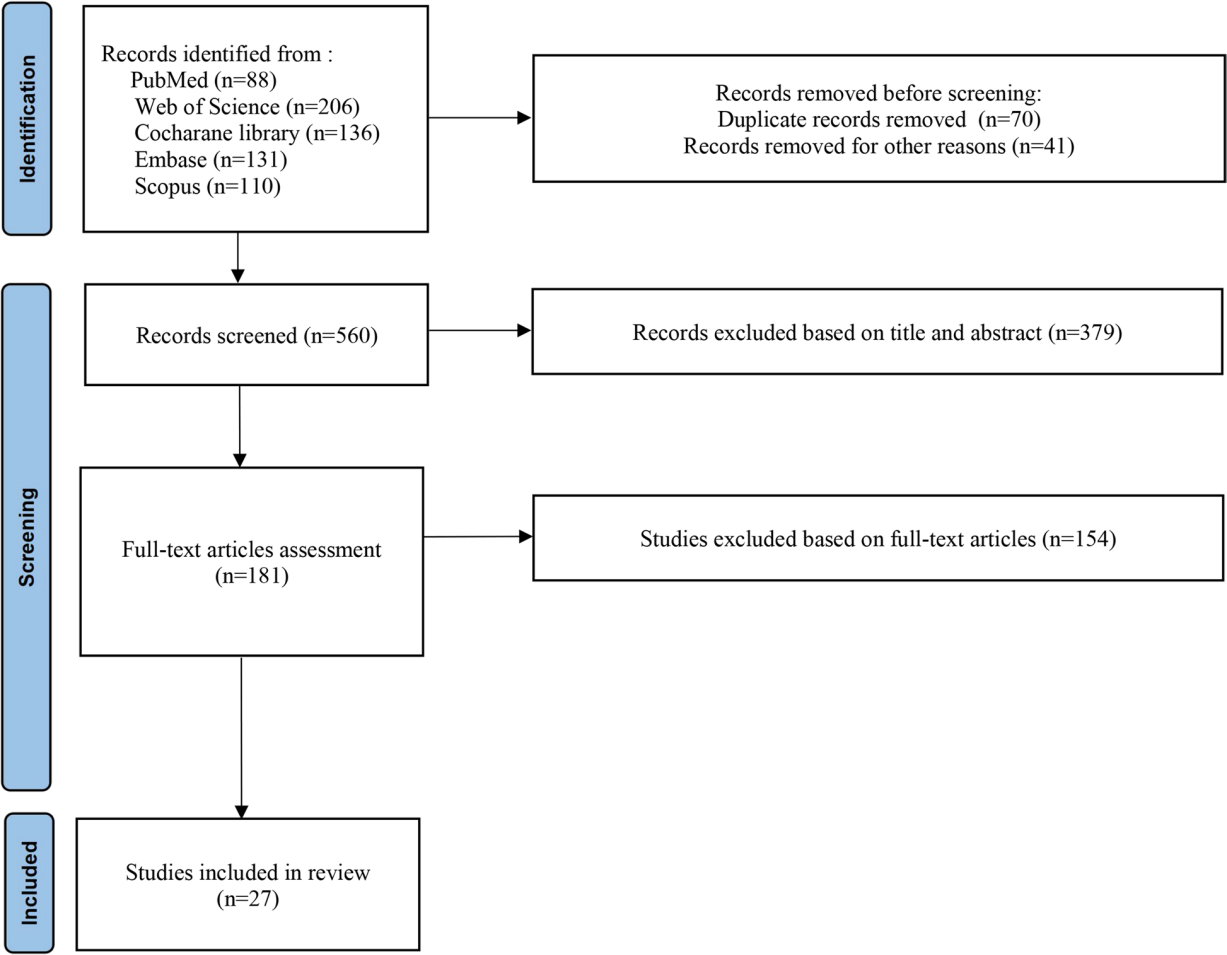
PRISMA flow diagram illustrating literature search and selection

The inclusion criteria of this meta-analysis were: (i) Studies analyzing patients diagnosed with LVT. (ii) Comparison of DOACs and VKAs regarding efficacy and/or safety outcomes stipulated above. The study selection was conducted independently by two authors. No restrictions were made with respect to the language of publications. Case reports, case series, and reviews were excluded. Repeated reports from the same cohorts or institutions were excluded to avoid duplication. Disagreements on study selection were resolved by discussion with the principal investigator (JZ). The PRISMA flow diagram in Fig. 1 illustrates the study selection process.

### Data extraction and quality assessment

Data extraction was conducted by two independent investigators using a format designed in advance. The following data were extracted: author, year, region, design, sample size, demographic characteristics of patients, follow-up duration, anticoagulation therapy, antiplatelet therapy, etiology of LVT, imaging modality, LVT area, left ventricular ejection fraction (LVEF), time in therapeutic range (TTR), thrombus resolution, ischemic stroke, systemic embolism, stroke/systemic embolism, all-cause mortality, adverse cardiovascular events, any bleeding, major bleeding, and intracranial hemorrhage. We attempted to contact the authors to obtain missing data; however, no response was received. The quality assessment was conducted independently by two authors. Quality of RCTs was evaluated using the Cochrane Collaboration Risk of Bias Tool (Supplemental Fig. 1). Observational studies were assessed based on the Newcastle–Ottawa scale (NOS). Observational studies with a score of ≥ 6 were considered high-quality. The overall score of the included studies varied from 5 to 8 points. The results are shown in Supplemental Table 2.

### Statistical analysis

The effect size was measured by risk ratio (RR) with 95% confidence interval (CI). Heterogeneity across studies was assessed using the I^2^ statistic. I^2^ greater than 50% was regarded as substantial heterogeneity. For analyses with low or moderate heterogeneity, the Mantel–Haenszel fixed effects model was utilized. If substantial heterogeneity was identified, the random effects model was employed. Forest plots were generated to examine the results visually. We performed subgroup analysis based on study design and the type of DOACs prescribed in individual studies. A sensitivity analysis was performed to evaluate the reliability of the results of individual studies and pooled analyses. Publication bias was evaluated by constructing funnel plots and conducting Egger’s test. A two-tailed *p*-value < 0.05 was considered statistically significant. All data analyses were conducted in Stata software version 15.1.

## Results

### Baseline findings

The demographic and clinical characteristics of the included studies are provided in Table 1. Twenty-seven studies [8, 9, 11–35] (four RCTs, one prospective observational study, and twenty-two retrospective cohort studies) with sample sizes ranging from 23 to 949 were analyzed in this study. All patients were allocated to the ‘DOACs’ or ‘VKAs’ group. The follow-up period of the individual studies ranged from 3 months to median 3.4 years. The selected studies were published between 2018 and 2023. The final selection included 11 studies from the United States of America [12, 13, 17, 21, 22, 25–30], 6 from Europe [11, 23, 24, 31–33], 7 from Asia [9, 14–16, 18, 20, 34], and 3 from Africa [8, 19, 35]. There was a higher overall proportion of male patients. AMI and ischemic cardiomyopathy were primary etiologies of LVT. Warfarin was most frequently administered in the VKAs cohort. The level of TTR was provided in only three publications. In the study by Youssef et al. [8], the INR time of the warfarin group within the TTR was 73%. In the RCT by Alcalai et al. [14], the average TTR in the warfarin group was 60%, with TTR > 65% in most patients and < 25% in two patients. In the study by Jones et al. [23], TTR was > 65% in 53.3% of patients who received VKAs. In terms of DOACs, three studies prescribed apixaban, three studies prescribed rivaroxaban, and the remaining studies used multiple types of DOACs. TTE was the most used imaging modality to detect LVT formation and monitor LVT resolution. Data regarding antiplatelet therapy was available in twenty studies [8, 9, 11, 13–15, 17, 18, 20–25, 28–31, 33, 35]. The rates and regimens (single or dual) of antiplatelet varied significantly between the included studies. In two studies [14, 20], all patients received dual antiplatelet therapy (DAPT). In an RCT by Youssef et al. [8], 16.0% of patients in the DOACs group and 20.0% in the VKAs group were prescribed a single antiplatelet medication, and the remaining patients received DAPT. In an RCT by Abdelnabi et al., the rate of DAPT was 53.1% [35]. The specific details of antiplatelet therapy are presented in Table 1.

**Table 1:** Characteristics of included studies

Author	Region	Design	Size (n)	Age (yrs)	Male (%)	MI (%)	ICM (%)	DCM (%)	DOACs, n	VKAs, type	DOACs, type	LVT area (cm^2^)	Mean LVEF (%)	Follow-up duration	Imaging modality	TTR > 65%	Antiplatelet therapy (%)
Youssef [8] 2023	Africa	RCT	50	DOACs: 52 ± 8.2 VKAs: 53 ± 7.9	N/A	DOACs: 44.0 VKAs: 40.0	N/A	N/A	25	warfarin	A	DOACs: 2.86 ± 0.96 × 1.54 ± 0.71 VKAs: 2.56 ± 1.15 × 1.59 ± 0.64	DOACs: 26.4 VKAs: 27.3	6 mths	TTE	73%	DOACs: Single (16.0) DAPT (80.0) VKAs: Single (20.0) DAPT (80.0)
Seiler [11] 2023	Europe	retrospective cohort study	101	DOACs: 64.3 ± 12.1 VKAs: 62.2 ± 14.2	DOACs: 87.5 VKAs: 77.4	DOACs: 54.2 VKAs: 47.1	DOACs: 87.5 VKAs: 90.6	DOACs: 4.2 VKAs: 0	48	Phenprocoumon	A = 17 R = 31	DOACs: 2.09 (1.05, 3.71) VKAs: 2.31 (1.6, 5.7)	DOACs: 40 ± 14 VKAs: 34 ± 11	median 26.6 mths	TTE	N/A	DOACs: Aspirin (37.5) Ticagrelor (4.2) Clopidogrel (41.7) Prasugrel (2.1) VKAs: Aspirin (50.9) Ticagrelor (3.8) Clopidogrel (36.0) Prasugrel (3.8)
Yang [9] 2023	Asia	retrospective registry study	124	50.2 ± 14.6	87.9	N/A	47.6	33.1	91	warfarin	N/A	2.2 (1.5, 3.2) × 1.2 (0.9, 1.7)	30 (22, 40)	> 3 mths	TTE, CMR	N/A	41.9 Single (25.0) DAPT (16.9)
Tamimi [12] 2022	USA	retrospective cohort study	191	mean 64.0	77.0	N/A	66.4	N/A	48	warfarin	A, E, R	N/A	N/A	N/A	echocardiography	N/A	N/A
Herald [13] 2022	USA	retrospective cohort study	433	DOACs: 66 (57, 75) VKAs: 65 (55, 73)	DOACs: 86.6 VKAs: 80.9	N/A	N/A	N/A	134	warfarin	A = 20 D = 108 R = 6	N/A	N/A	3.4 yrs (1.9–5.9)	TTE	N/A	DOACs: 44.8 VKAs: 37.8
Alcalai [14] 2022	Asia	RCT	35	DOACs: 55.5 ± 12.9 VKAs: 58.8 ± 10.2	DOACs: 72.2 VKAs: 88.2	100.0	DOACs: 22.2 VKAs: 17.7	N/A	18	warfarin	A	DOACs: 1.99 ± 0.94 × 1.24 ± 0.58 VKAs: 1.85 ± 0.69 × 1.23 ± 0.40	DOACs: 35.0 ± 5.0 VKAs: 36.0 ± 7.0	3 mths	TTE	N/A	DAPT (100.0)
Zhang [20] 2022	Asia	retrospective cohort study	64	DOACs: 60.3 ± 14.7 VKAs: 61.3 ± 9.0	DOACs: 72.7 VKAs: 74.2	100.0	N/A	N/A	33	warfarin	R	N/A	DOACs: 42.9 ± 13.1 VKAs: 41.4 ± 10.8	median 25.0 mths	TTE	N/A	DAPT (100.0)
Abdelnabi [35] 2021	Africa	RCT	79	49.6 ± 12.5	57	N/A	78.5	N/A	39	warfarin	R	1.61 ± 0.438 × 1.15 ± 0.264	36.6	6 mths	TTE	N/A	DAPT (53.1)
Albabtain [15] 2021	Asia	retrospective cohort study	63	DOACs: 58.25 (17.73) VKAs: 59 (15.62)	DOACs: 85.71 VKAs: 97.14	DOACs: 57.14 VKAs: 71.43	N/A	N/A	28	warfarin	R	DOACs: 1.75 (0.7–3.49) VKAs: 1.77 (0.66–2.21)	DOACs: 26.43 (8.15) VKAs: 27.29 (7.8)	DOACs: 9.5 (6–32.5) mths VKAs: 14(3–41) mths	TTE	N/A	DOACs: Aspirin (67.86) Clopidogrel (46.43) VKAs: Aspirin (57.14) Clopidogrel (60.0)
Mihm [17] 2021	USA	retrospective cohort study	108	DOACs: 63.3 (14.4) VKAs: 60.3 (13.9)	DOACs: 69.7 VKAs: 72.0	DOACs: 15.2 VKAs: 14.7	N/A	N/A	33	warfarin	A = 23 R = 10	N/A	DOACs: 32.58 (16.4) VKAs: 27.95 (12.6)	6 mths	TTE, CMR	N/A	DOACs: Aspirin (57.6) P2Y12i (21.2) VKAs: Aspirin (73.3) P2Y12i (25.3)
Xu [18] 2021	Asia	retrospective cohort study	87	DOACs: 59.4 ± 11.5 VKAs: 61.9 ± 12.2	DOACs: 76.0 VKAs: 75.8	DOACs: 16.0 VKAs: 21.0	DOACs: 72.0 VKAs: 77.4	N/A	25	warfarin	D = 9 R = 16	N/A	DOACs: 33.8 ± 5.7 VKAs: 37.6 ± 6.6	2.37 ± 2.1 yrs	TTE	N/A	DOACs: 44.0 VKAs: 43.5
Varwani [19] 2021	Africa	retrospective cohort study	100	mean 60.9	77.0	28.0	42.0	N/A	58	warfarin	A = 5 D = 7 R = 46	N/A	28.5 ± 11.0	N/A	TTE	N/A	N/A
Bass [21] 2021	USA	retrospective cohort study	949	DOACs: 63.4 ± 16.7 VKAs: 61.6 ± 15.3	DOACs: 69.4 VKAs: 70.9	DOACs: 42.8 VKAs: 57.6	N/A	N/A	180	warfarin	A = 79 R = 77 D = 29	N/A	N/A	3 mths	N/A	N/A	DOACs: 46.7 VKAs: 55.7
Cochran [27] 2021	USA	retrospective cohort study	73	DOACs: 51.5 (39.0–73.0) VKAs: 62.0 (34.0–84.0)	DOACs: 78.6 VKAs: 76.3	DOACs: 43.0 VKAs: 49.0	N/A	N/A	14	warfarin	A, E, D, R	N/A	N/A	12 mths	TTE	N/A	N/A
Jones [23] 2021	Europe	prospective cohort study	101	DOACs: 58.73 ± 14.2 VKAs: 60.81 ± 14.3	DOACs: 80.4 VKAs: 85.0	100.0	N/A	N/A	41	warfarin	A = 15 E = 2 R = 24	N/A	DOACs: 33.5 ± 10.0 VKAs: 35.4 ± 9.0	median 2.2 yrs	TTE, CMR	53.3%	DOACs: 92.7 Single (24.4) DAPT (68.3) VKAs: 91.7 Single (21.7) DAPT (70.0)
Willeford [25] 2021	USA	retrospective cohort study	151	DOACs: 54 (48–64) VKAs: 56 (49–65.5)	DOACs: 77.3 VKAs: 80.6	DOACs: 22.7 VKAs: 26.4	N/A	N/A	22	warfarin	A = 4 R = 18	N/A	N/A	median: 254 days	echocardiography	N/A	56.3 Single (37.1) DAPT (19.2)
Minciunescu [22] 2020	USA	retrospective cohort study	212	DOACs: 60.4 ± 15.9 VKAs: 59.5 ± 13.9	DOACs: 80.7 VKAs: 75.5	N/A	N/A	N/A	57	warfarin	N/A	N/A	N/A	N/A	N/A	N/A	74.5
Iqbal [24] 2020	Europe	retrospective cohort study	84	DOACs: 62 ± 13 VKAs: 62 ± 14	DOACs: 91.0 VKAs: 89.0	N/A	DOACs: 82.0 VKAs: 89.0	DOACs: 9.0 VKAs: 3.0	22	warfarin	A = 8 D = 1 R = 13	N/A	DOACs: 31 ± 13 VKAs: 35 ± 13	3.0 ± 1.4 yrs	TTE, TEE, CMR	N/A	65.0 Single (27.0) DAPT (38.0)
Yunis [26] 2020	USA	retrospective cohort study	264	N/A	N/A	N/A	N/A	N/A	64	warfarin	N/A	N/A	N/A	24 mths	TTE, TEE	N/A	N/A
Isa [16] 2020	Asia	RCT	27	DOACs: 55.36 (11.04) VKAs: 55.00 (11.42)	DOACs: 92.9 VKAs: 92.3	N/A	DOACs: 64.3 VKAs: 61.5	N/A	14	warfarin	A	DOACs: 1.98 (0.41–3.55) VKAs: 0.95 (0.17–1.73)	33.5 ± 5.73	3 mths	TTE	N/A	N/A
Ali [30] 2020	USA	retrospective cohort study	92	DOACs: 59.2 ± 11.9 VKAs: 58.0 ± 16.3	DOACs: 81.3 VKAs: 81.7	N/A	N/A	N/A	32	warfarin	A = 13 R = 18 D = 1	N/A	DOACs: 23.0 ± 9.4 VKAs: 23.2 ± 11.2	> 1yr in 58.2%	TTE, CMR	N/A	Aspirin: 65.45Clopidogrel: 14.55Ticagrelor: 0.91Prasugrel: 1.82
Robinson [28] 2020	USA	retrospective cohort study	514	DOACs: 58.1 (14.9) VKAs: 58.2 (15.1)	DOACs: 77.7 VKAs: 72.0	N/A	DOACs: 54.5 VKAs: 62.7	N/A	121	warfarin	N/A	DOACs: 2.8 (2.1) VKAs: 2.8 (2.5)	DOACs: 27.7 (13.8) VKAs: 28.2 (12.4)	351 days (51–866)	TTE	N/A	DOACs: 63.6 VKAs: 69.5
Guddeti [29] 2020	USA	retrospective cohort study	99	DOACs: 60.7 ± 13.1 VKAs: 61.3 ± 12.2	DOACs: 79.0 VKAs: 68.8	DOACs: 21.0 VKAs: 20.5	DOACs: 52.6 VKAs: 60	N/A	19	warfarin	A = 15 R = 2 D = 2	N/A	DOACs: 25(20–40) VKAs: 25(20–35)	10.4 ± 3.4 mths	echocardiography	N/A	DOACs: Aspirin (57.9) P2Y12i (15.8) VKAs: Aspirin (67.5) P2Y12i (15.0)
Daher [31] 2020	Europe	retrospective cohort study	59	DOACs: 57.0 ± 14.0 VKAs: 61.0 ± 13.0	DOACs: 82.4 VKAs: 83.0	N/A	DOACs: 88.0 VKAs: 74.0	13.5	17	warfarin, acenocoumarol, fluindione	A = 12 R = 4 D = 1	N/A	DOACs: 41.0 ± 8.0 VKAs: 36.0 ± 12.0	N/A	TTE	N/A	DOACs: Aspirin (58.8) P2Y12i (64.7) VKAs: Aspirin (66.7) P2Y12i (40.5)
Lim [34] 2019	Asia	retrospective cohort study	23	55 ± 9.6	73.9	N/A	87.0	N/A	5	warfarin	D = 3 R = 2	N/A	30.8 ± 10.6	N/A	echocardiography	N/A	N/A
Gama [32] 2019	Europe	retrospective cohort study	66	69 ± 12	77.3	N/A	N/A	N/A	13	warfarin	N/A	N/A	N/A	N/A	echocardiography, CMR	N/A	N/A
Jaidka [33] 2018	Europe	retrospective cohort study	49	DOACs: 57.2 ± 9.3 VKAs: 61.3 ± 12.1	DOACs: 75.0 VKAs: 75.7	100.0	N/A	N/A	12	warfarin	N/A	N/A	DOACs: 36.7 ± 10.1 VKAs: 20.0 ± 20.7	6 mths	TTE	N/A	DOACs:Aspirin (75) Clopidogrel (100.0) Ticagrelor (0.0) VKAs:Aspirin (89.9) Clopidogrel (89.9) Ticagrelor (8.1)

*Abbreviations*: *RCT* Randomized controlled trial, *Yrs *Years, *N/A* Not available, *MI* Myocardial infarction, *DOACs* Direct oral anticoagulants, *VKAs* Vitamin K antagonists, *ICM* Ischemic cardiomyopathy, *DCM* Dilated cardiomyopathy, *A* Apixaban, *E* Edoxaban, *D* Dabigatran, *R* Rivaroxaban, *LVT*, Left ventricular thrombus, *LVEF* Left ventricular ejection fraction, *Mths* Months, *TTE* Transthoracic echocardiography, *TTR* Time in therapeutic range, *DAPT* Dual antiplatelet therapy, *CMR* Cardiac magnetic resonance, *TEE* Transesophageal echocardiography, *P2Y12i* P2Y12 inhibitor

### LVT resolution

**Fig. 2:**
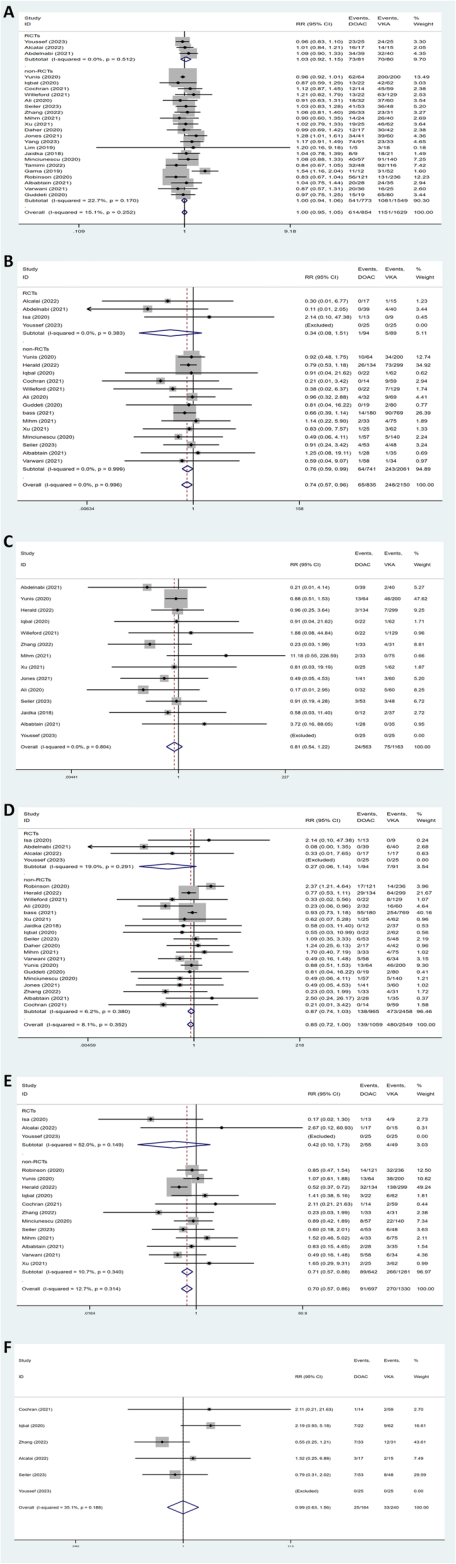
Forest plots for efficacy outcomes of DOACs and VKAs in the treatment of LVT. **A** thrombus resolution. **B** ischemic stroke. **C** systemic embolism. **D** stroke/systemic embolism. **E** all-cause mortality. **F** adverse cardiovascular events

Three RCTs, one prospective study, and twenty retrospective studies reported LVT resolution outcomes (Fig. 2A). There was no difference regarding LVT resolution between the DOACs and VKAs groups (RR = 1.00, 95% CI 0.95–1.05, *P* = 0.99, I^2^ = 15.1%). The subgroup analysis based on study type showed similar results. Compared to the VKAs group, there were similar rates of thrombus resolution in the DOACs group (Supplementary Fig. 2A, B) at 3 months (RR = 1.11, 95% CI 0.97–1.27, P = 0.12, I^2^ = 0.0%) and 6 months (RR = 1.04, 95% CI 0.84–1.28, *P* = 0.72, I^2^ = 56.0%).

### Stroke

Incidence of stroke was available in four randomized and fourteen retrospective studies. As shown in Fig. 2B, DOACs were associated with a significantly lower risk of stroke (RR = 0.74, 95% CI 0.57–0.96, *P* = 0.021, I^2^ = 0.0%). In a subgroup analysis including only RCTs, no significant difference was observed (RR = 0.34, 95% CI 0.08–1.51, *P* = 0.15, I^2^ = 0.0%). Subgroup analysis of non-RCTs showed significant results, with the pooled analysis exhibiting lower risk in the DOACs arm (RR = 0.76, 95% CI 0.59–0.99, *P* = 0.04, I^2^ = 0.0%).

### Systemic embolism

As illustrated in Fig. 2C, a total of fourteen studies assessed the efficacy of systemic embolism prevention. No significant difference in the incidence of systemic embolism was found between the two groups (RR = 0.81, 95% CI 0.54–1.22, *P* = 0.32, I^2^ = 0.0%).

### Stroke/systemic embolism

As presented in Fig. 2D, the composite endpoint of stroke and systemic embolism were assessed in twenty-three studies. The meta-analysis showed no difference between the two therapies (RR = 0.85, 95% CI 0.72–1.00, *P* = 0.056, I^2^ = 8.1%). No significant differences were observed in RCTs or non-RCTs subgroups.

### All-cause mortality

Data regarding all-cause mortality were available from fifteen publications. The overall meta-analysis demonstrated that DOACs were significantly associated with a lower incidence of all-cause mortality than VKAs (RR = 0.70, 95% CI 0.57–0.86, *P* = 0.001, I^2^ = 12.7%). There was also a significant difference between DOACs and VKAs in the subgroup analysis for non-RCTs (RR = 0.71, 95% CI 0.57–0.88, *P* = 0.001, I^2^ = 10.7%). Figure 2E illustrates the results.

### Adverse cardiovascular events

Figure 2F presents the forest plot for adverse cardiovascular events. The occurrence of adverse cardiovascular events did not differ significantly between the two anticoagulation therapies (RR = 0.99, 95% CI 0.63–1.56, *P* = 0.92, I^2^ = 35.1%).

### Any bleeding

**Fig. 3:**
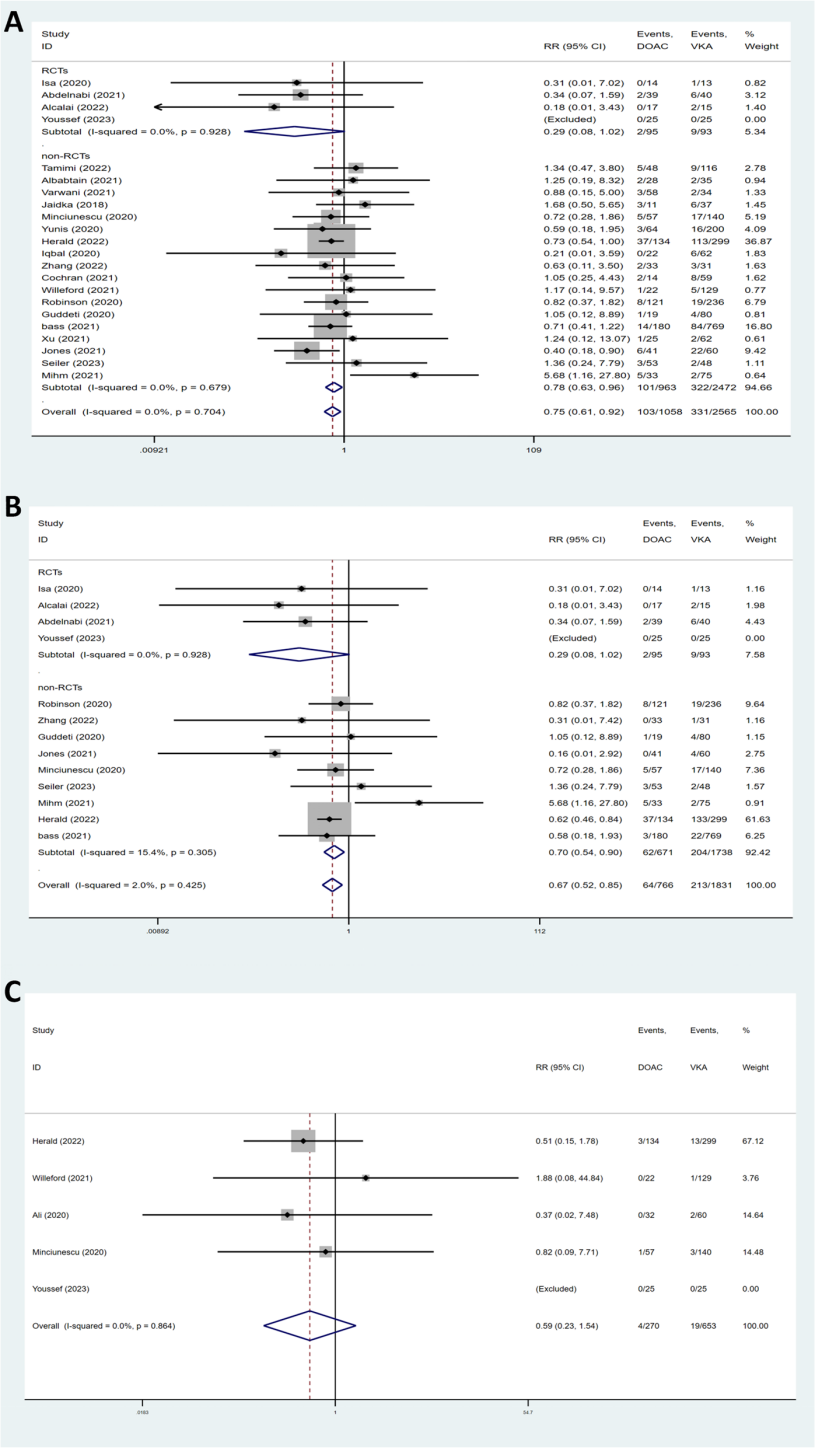
Forest plots for safety outcomes of DOACs and VKAs in the treatment of LVT. **A** any bleeding. **B** major bleeding. **C** intracranial hemorrhage

Twenty-two studies provided data on any bleeding (Fig. 3A). According to the meta-analysis, bleeding event rates were significantly lower in the DOACs group (RR = 0.75, 95% CI 0.61–0.92, *P* = 0.006). There was no heterogeneity among the studies (I^2^ = 0.0%). Subgroup analysis also revealed that there was a significant difference in non-RCT subgroups (RR = 0.78, 95% CI 0.63–0.96, *P* = 0.017, I^2^ = 0.0%).

### Major bleeding

Major bleeding events were reported by thirteen studies. As presented in Fig. 3B, pooled summary using the fixed-effect model suggested that patients receiving DOACs had a lower risk of major bleeding than those who were prescribed with VKAs (RR = 0.67, 95% CI 0.52–0.85, *P* = 0.001, I^2^ = 2.0%). In the RCT subgroup analysis, outcomes of major bleeding were not significantly different between the two groups (RR = 0.29, 95% CI 0.08–1.02, *P* = 0.054, I^2^ = 0.0%).

### Intracranial hemorrhage

Intracranial hemorrhage was reported in five studies (Fig. 3C). The meta-analysis showed no significant difference regarding intracranial hemorrhage between the DOACs and VKAs (RR = 0.59, 95% CI 0.23–1.54, *P* = 0.28). There was no heterogeneity across the studies (I^2^ = 0.0%).

### Subgroup analysis for DOACs type

Six publications were included in the subgroup analysis for apixaban (Supplementary Fig. 3A-C). There were no significant differences regarding LVT resolution (RR = 1.14, 95% CI 0.93–1.39, *P* = 0.21, I^2^ = 75.9%), stroke/systemic embolism (RR = 0.70, 95% CI 0.46–1.05, *P* = 0.085, I^2^ = 0.0%) and bleeding events (RR = 0.53, 95% CI 0.23–1.21, *P* = 0.13, I^2^ = 0.0%) between apixaban and the VKAs group. Meta-analysis showed that rivaroxaban was associated with lower risk of stroke (RR = 0.24, 95% CI 0.08–0.72, *P* = 0.011, I^2^ = 0.0%). Outcomes of thrombus resolution (RR = 1.13, 95% CI 1.00–1.28, *P* = 0.056, I^2^ = 0.0%), stroke/systemic embolism (RR = 0.41, 95% CI 0.07–2.30, *P* = 0.31, I^2^ = 66.2%), any bleeding (RR = 0.89, 95% CI 0.52–1.53, *P* = 0.68, I^2^ = 0.0%) and major bleeding (RR = 0.51, 95% CI 0.17–1.52, *P* = 0.22, I^2^ = 0.0%) were comparable between rivaroxaban and VKAs. Supplementary Fig. 4A-E illustrates the forest plots for this comparison.

### Publication bias assessment

Egger’s test showed no evident publication bias in LVT resolution (*P* = 0.13), stroke (*P* = 0.33), systemic embolism (*P* = 0.86), stroke/systemic embolism (*P* = 0.18), all-cause mortality (*P* = 0.25), any bleeding (*P* = 0.66) and major bleeding (*P* = 0.92). Supplementary Fig. 5A-G presents the corresponding funnel plots.

### Sensitivity analysis

We conducted a sensitivity analysis for the primary analyses (Supplementary Fig. 6). The meta-analysis result of LVT resolution, ischemic stroke, systemic embolism, stroke/systemic embolism, any bleeding, major bleeding, intracranial hemorrhage, all-cause mortality, and adverse cardiovascular events did not significantly change.

## Discussion

The primary objective of this systematic review and meta-analysis was to compare the efficacy and safety profile of DOACs versus VKAs in the treatment of LVT. To the best of our knowledge, this is the meta-analysis study with the greatest sample size. Our meta-analysis found that DOACs are safer and equally effective compared to VKAs in the treatment of LVT.

LVT most often occurs after acute anterior STEMI, followed by ischemic cardiomyopathy and nonischemic cardiomyopathies such as DCM, hypertrophic cardiomyopathy (HCM), Takotsubo cardiomyopathy, and cardiac amyloidosis [1, 31, 36, 37]. In patients who develop LVT after AMI, the risk for embolism is 5.5-fold higher than those with no cardiac thrombosis [38].

The well-established Virchow’s triad demonstrates that stasis, endothelial injury, and hypercoagulation are three primary prerequisites for venous thromboembolism [39]. The same principle is applied to the pathophysiology of LVT formation. Low LVEF is an independent risk factor for LVT both in the settings of AMI and DCM [40]. Inflammatory response, hypercoagulation, and endocardial injuries have also been identified as critical drivers of LVT formation [1, 41, 42].

In clinical practice, TTE is the primary imaging modality for detecting LVT. However, late gadolinium enhancement (LGE) CMR is the gold standard for the visualization of LVT as it has better diagnostic sensitivity than TTE, especially for mural or small volume thrombus [43, 44]. The contrast-enhanced ultrasound can be used to improve the sensitivity of echocardiogram in detecting LVT formation [45]. There is little evidence to support the notion that using CMR to identify LVT rather than ultrasonography is associated with a better outcome. In cases where LVT formation is clinically suspected but cannot be verified by echocardiography, it is proposed that CMR is necessary [1].

A meta-analysis by Vaitkus et al. [38] demonstrated that patients with LVT and MI could see a reduction in the risk of embolic events with adequate anticoagulation. The optimal anticoagulation regimen for LVT has not yet been fully established. The current clinical guidelines still recommend warfarin as the preferred anticoagulation agent for this specific population. Although warfarin is clinically effective, it has inherent limitations, including a narrow therapeutic window, frequent monitoring of coagulation function, susceptibility to drugs and food, long half-lives, and low therapy adherence [46].

Previous studies have observed that DOACs share a comparable therapeutic efficacy and safety with VKAs in the resolution of deep venous and left atrial appendage thrombus [47–49]. In the past decade, numerous retrospective cohort studies have explored the off-label use of DOACs in the resolution of LVT, but they have shown inconsistent results [24, 25, 31, 50]. Four RCTs [8, 14, 16, 35] have shown that DOACs (apixaban and rivaroxaban) have a non-inferior benefit/risk profile to VKAs in the treatment of LVT, but the length of the follow-up period was limited to 3 to 6 months. Consistent with the previous meta-analysis studies [27, 51–53], our study found that there was no significant difference in terms of LVT resolution, risks of systemic embolism, and stroke/systemic embolism between the two anticoagulation therapies. In contrast to our findings, Burmeister et al. [44] suggested that DOACs were associated with a significantly higher rate of LVT resolution than VKAs.

Our meta-analysis showed that DOACs were associated with significantly lower incidence of stroke (RR = 0.74, 95% CI 0.57–0.96, *P* = 0.021), all-cause mortality (RR = 0.70, 95% CI 0.57–0.86, *P* = 0.001), any bleeding (RR = 0.75, 95% CI 0.61–0.92, *P* = 0.005) and major bleeding (RR = 0.67, 95% CI 0.52–0.85, *P* = 0.001). The selection of included studies can partly explain these discrepancies between prior investigations.

We also performed a subgroup analysis according to DOAC type. Meta-analysis showed that the risk of stroke (RR = 0.24, 95% CI 0.08–0.72, *P* = 0.011) was significantly lower in patients administered rivaroxaban. We observed similar efficacy regarding LVT resolution between VKAs and rivaroxaban or apixaban.

The duration of anticoagulants prescribed for LTV remains to be determined. The embolic risk has been reported to be highest in the first two weeks after MI, and the risk of LVT recurrence is highest in the first three months following MI [54]. The most recent scientific statement from the AHA recommends reimaging at 3 months after AMI, and it was suggested that anticoagulation therapy should be discontinued if no thrombus is detectable [1]. Regarding patients with DCM and LVT, the anticoagulation duration is suggested for at least 3 to 6 months [1, 55]. Based on available data, our meta-analysis showed that the resolution of LVT at 3 or 6 months did not differ significantly between DOACs and VKAs.

In summary, the current meta-analysis supports the use of DOACs for the treatment of LVT. However, more robust data from large randomized trials with adequate sample size and follow-up length is still required.

There are several limitations of this meta-analysis. First, our findings are primarily based on retrospective cohorts with varied follow-up duration and are therefore susceptible to various biases. Ultrasound was the primary imaging technique utilized to confirm the LVT resolution in the included studies. Hence, the complete thrombus resolution was possibly overestimated. Additionally, there was heterogeneity in the endpoint definitions of major bleeding events between the individual studies. Eight of the selected papers were published as non-full text. TTR, an essential influencing factor of efficacy and safety of VKAs, was not systematically measured in the majority of included studies. It was uncertain whether the superiority of DOACs over VKAs could be partly attributed to poor maintenance of therapeutic TTR. Generally, a combination of anticoagulation therapy and antiplatelet drugs predisposes patients to bleeding complications. Regarding antiplatelet regimens, there were significant differences between the included studies. Given the limited data, we did not perform further analysis to assess the effects of anticoagulation alone versus anticoagulation plus antiplatelet agents. The optimal dose of DOACs in the treatment of LVT was not explored in this meta-analysis. Future studies should compare the clinical effects of standard doses versus low doses of DOACs. Taking into account these limitations, the findings of our study should be interpreted with caution.

## Conclusion

In our study, DOACs demonstrated non-inferior effectiveness and superior safety to VKAs in the treatment of LVT. DOACs may be a feasible choice for this patient population. Anticoagulation therapy should be individualized in patients with LVT, and clinical decisions should be made after a full discussion between patients and physicians. More robust large RCTs are required to investigate the optimal regimen and duration of anticoagulation in the management of LVT.

### Supplementary Information

**Additional file 1: Supplementary Table 1.** The detailed search strategy of databases. **Supplementary Table 2.** Quality assessment of included observational studies using the Newcastle-Ottawa Scale. **Supplementary Figure 1.** Quality assessment of the included randomized controlled trials. **Supplementary Figure 2.** Forest plot comparing the efficacy of DOACs and VKA in the treatment of LVT. (A) after 3 months of treatment. (B) after 6 months of treatment. **Supplementary Figure 3.** Forest plot to compare apixaban with VKAs in outcomes including (A) LVT resolution, (B) stoke/systemic embolism, (C) any bleeding. **Supplementary Figure 4.** Forest plot to compare rivaroxaban with VKAs in outcomes including (A) LVT resolution. (B) stroke. (C) stroke/systemic embolism. (D) major bleeding. (E) any bleeding. **Supplementary Figure 5.** The funnel plots based on the outcomes. (A) funnel plot for LVT resolution. (B) funnel plot for stroke. (C) funnel plot for systemic embolism. (D) funnel plot for stroke/systemic embolism. (E) funnel plot for all-cause mortality. (F) funnel plot for any bleeding. (G)funnel plot for major bleeding events. **Supplementary Figure 6.** Results of sensitivity analyses. (A) LVT resolution. (B) ischemic stroke. (C) systemic embolism. (D) stroke/systemic embolism. (E) any bleeding. (F) major bleeding. (G) intracranial hemorrhage. (H) all-cause mortality. (I) adverse cardiovascular events.

## Data Availability

The data is available from the corresponding author.

## Abbreviations

LVT: Left ventricular thrombus
AMI: Acute myocardial infarction
VKAs: Vitamin K antagonists
RCTs: Randomized controlled trials
STEMI: ST-segment elevation myocardial infarction
DOACs: Direct oral anticoagulants
DCM: Dilated cardiomyopathy
AHA: American Heart Association
PRISMA: Preferred Reporting Items for Systematic Reviews and Meta-Analyses
TTE: Transthoracic echocardiography
TEE: Transesophageal echocardiography
CMR: Cardiac magnetic resonance
LVEF: Left ventricular ejection fraction
NOS: Newcastle–Ottawa scale
RR: Risk ratio
CI: Confidence interval
HCM: Hypertrophic cardiomyopathy
LGE: Late gadolinium enhancement
DAPT: Dual antiplatelet therapy
TTR: Time in therapeutic range
