# Supplementary material for: Comparative effectiveness and safety of DOACs vs. VKAs in treatment of left ventricular thrombus- a meta-analysis update
Source: Thromb J. 2024 Mar 1;22:23. doi: 10.1186/s12959-024-00585-9 (PMC10905864; doi:10.1186/s12959-024-00585-9)

**Supplementary Data**

**Supplementary Legends:**

***Supplementary Table 1.*** The detailed search strategy of databases.

***Supplementary Table 2.*** Quality assessment of included observational studies using the Newcastle-Ottawa Scale.

***Supplementary Figure 1.*** Quality assessment of the included randomized controlled trials.

***Supplementary Figure 2.*** Forest plot comparing the efficacy of DOACs and VKA in the treatment of LVT. (A) after 3 months of treatment. (B) after 6 months of treatment.

***Supplementary Figure 3.*** Forest plot to compare apixaban with VKAs in outcomes including (A) LVT resolution, (B) stoke/systemic embolism, (C) any bleeding.

***Supplementary Figure 4.***  Forest plot to compare rivaroxaban with VKAs in outcomes including (A) LVT resolution. (B) stroke. (C) stroke/systemic embolism. (D) major bleeding. (E) any bleeding.

***Supplementary Figure 5.*** The funnel plots based on the outcomes. (A) funnel plot for LVT resolution. (B) funnel plot for stroke. (C) funnel plot for systemic embolism. (D) funnel plot for stroke/systemic embolism. (E) funnel plot for all-cause mortality. (F) funnel plot for any bleeding. (G)funnel plot for major bleeding events.

***Supplementary Figure 6.*** Results of sensitivity analyses. (A) LVT resolution. (B) ischemic stroke. (C) systemic embolism. (D) stroke/systemic embolism. (E) any bleeding. (F) major bleeding. (G) intracranial hemorrhage. (H) all-cause mortality. (I) adverse cardiovascular events.

***Supplemental Table 1.*** The detailed search strategy of databases.

| **Database** | **Search strategy** | **Number** |
| --- | --- | --- |
| PubMed | (left ventricular thrombus[Title] OR left ventricular thrombi[Title]) AND (anticoagulation[Title] OR warfarin[Title] OR vitamin K antagonist[Title] OR non-vitamin K antagonist[Title] OR direct oral anticoagulant[Title] OR novel oral anticoagulant[Title] OR rivaroxaban[Title] OR apixaban[Title] OR edoxaban[Title] OR dabigatran[Title]) | 88 |
| Web of Science | left ventricular thrombus or left ventricular thrombi(Title) and anticoagulation or warfarin or vitamin K antagonist or non-vitamin K antagonist or direct oral anticoagulant or novel oral anticoagulant or rivaroxaban or apixaban edoxaban or dabigatran (Title) | 206 |
| Embase | ('left ventricular thrombus':ti OR 'left ventricular thrombi':ti) AND (anticoagulation:ti OR warfarin:ti OR 'vitamin k antagonist':ti OR 'non-vitamin k antagonist':ti OR 'direct oral anticoagulant':ti OR 'novel oral anticoagulant':ti OR rivaroxaban:ti OR apixaban:ti OR edoxaban:ti OR dabigatran:ti) | 131 |
| Cochrane | left ventricular thrombus or left ventricular thrombi in Title Abstract Keyword AND anticoagulation or warfarin or vitamin K antagonist or non-vitamin K antagonist or direct oral anticoagulant or novel oral anticoagulant or rivaroxaban or apixaban edoxaban or dabigatran | 136 |
| Scopus | ( TITLE ( {left ventricular thrombus} OR {left ventricular thrombi} ) AND TITLE ( "anticoagulation" OR "warfarin" OR "vitamin K antagonist" OR {non-vitamin K antagonist} OR {direct oral anticoagulant} OR {novel oral anticoagulant} OR "rivaroxaban" OR "apixaban" OR "edoxaban" OR "dabigatran" ) ) | 110 |

***Supplementary Table 2.*** Quality assessment of included observational studies using the Newcastle-Ottawa Scale.

| **Study** | **Selection** | | | | **Comparability** | **Outcome** | | | **Score** |
| --- | --- | --- | --- | --- | --- | --- | --- | --- | --- |
|  | Representativeness of the exposed cohort | Selection of the non-exposed cohort | Ascertainment of exposure | Outcome of interest was not present at start of study | Comparability of cohorts on the basis of the design or analysis | Assessment of outcome | Adequate follow-up duration | Adequate follow-up rate |  |
| **Seiler**  **2023** | * | * | * | * | * | * | * | * | 8 |
| **Yang**  **2023** | * | * | * | * | * | * | * | * | 8 |
| **Tamimi**  **2022** | * |  | * | * |  | * | * |  | 5 |
| **Herald**  **2022** | * | * | * |  | * | * | * | * | 7 |
| **Zhang**  **2022** | * | * | * |  | * | * | * | * | 7 |
| **Albabtain**  **2021** | * | * | * | * | * | * | * | * | 8 |
| **Mihm**  **2021** | * | * | * |  | * | * | * | * | 7 |
| **Xu**  **2021** | * | * | * |  | * | * | * | * | 7 |
| **Varwani**  **2021** | * | * | * |  |  | * | * | * | 6 |
| **Jones**  **2021** | * | * | * |  | * | * | * | * | 7 |
| **Bass**  **2021** | * | * | * |  | * | * | * |  | 6 |
| **Cochran**  **2021** | * | * | * |  | * | * | * | * | 7 |
| **Willeford**  **2021** | * | * | * |  |  | * | * | * | 6 |
| **Minciunescu**  **2020** | * | * | * |  |  | * | * |  | 5 |
| **Iqbal**  **2020** | * | * | * |  | ** | * | * | * | 8 |
| **Yunis**  **2020** | * | * | * |  |  | * | * |  | 5 |
| **Ali**  **2020** | * | * | * |  |  | * | * |  | 5 |
| **Robinson**  **2020** | * | * | * | * |  | * | * | * | 7 |
| **Guddeti**  **2020** | * | * | * |  | ** | * | * | * | 8 |
| **Daher**  **2020** | * | * | * |  | * | * | * | * | 7 |
| **Lim**  **2019** | * | * | * |  |  | * | * |  | 5 |
| **Gama**  **2019** | * | * | * |  | * | * |  |  | 5 |
| **Jadika**  **2018** | * | * | * |  |  | * | * | * | 6 |


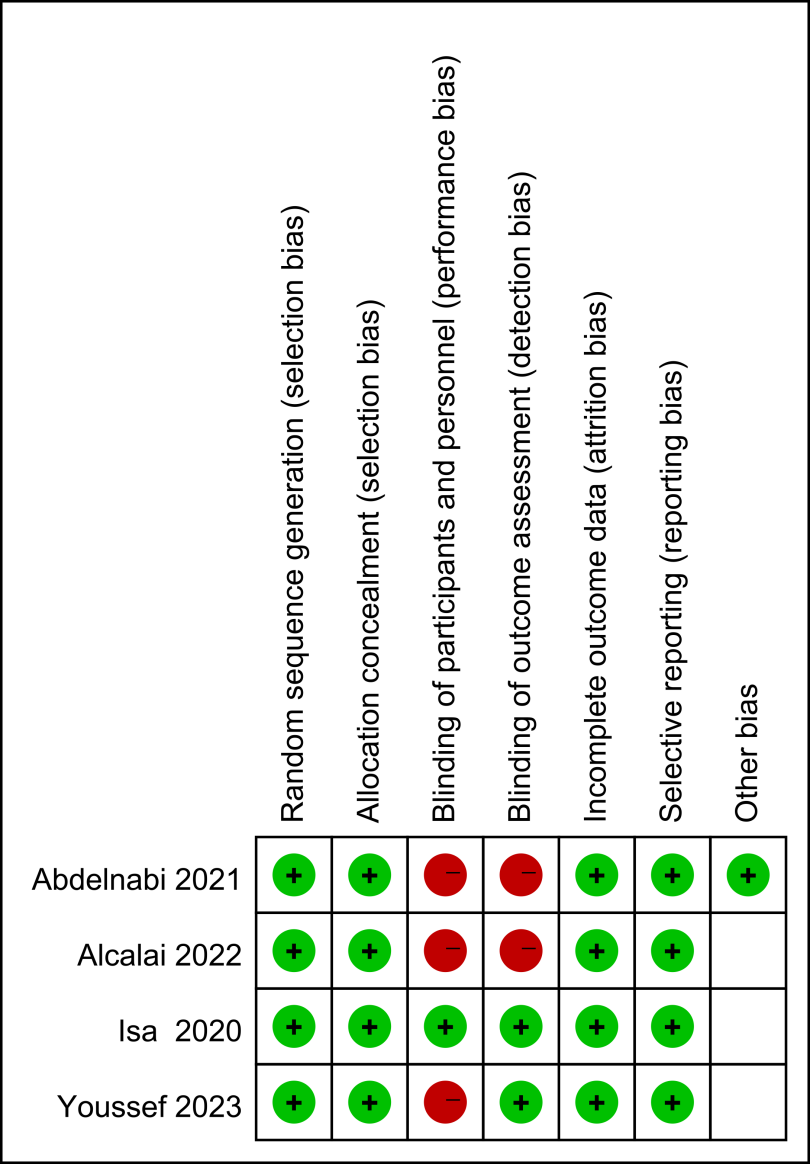


***Supplementary Figure 1.*** Quality assessment of the included randomized controlled trials.

***Supplementary Figure 2.*** Forest plot comparing the efficacy of DOACs and VKA in the treatment of LVT. (A) after 3 months of treatment. (B) after 6 months of treatment.

**
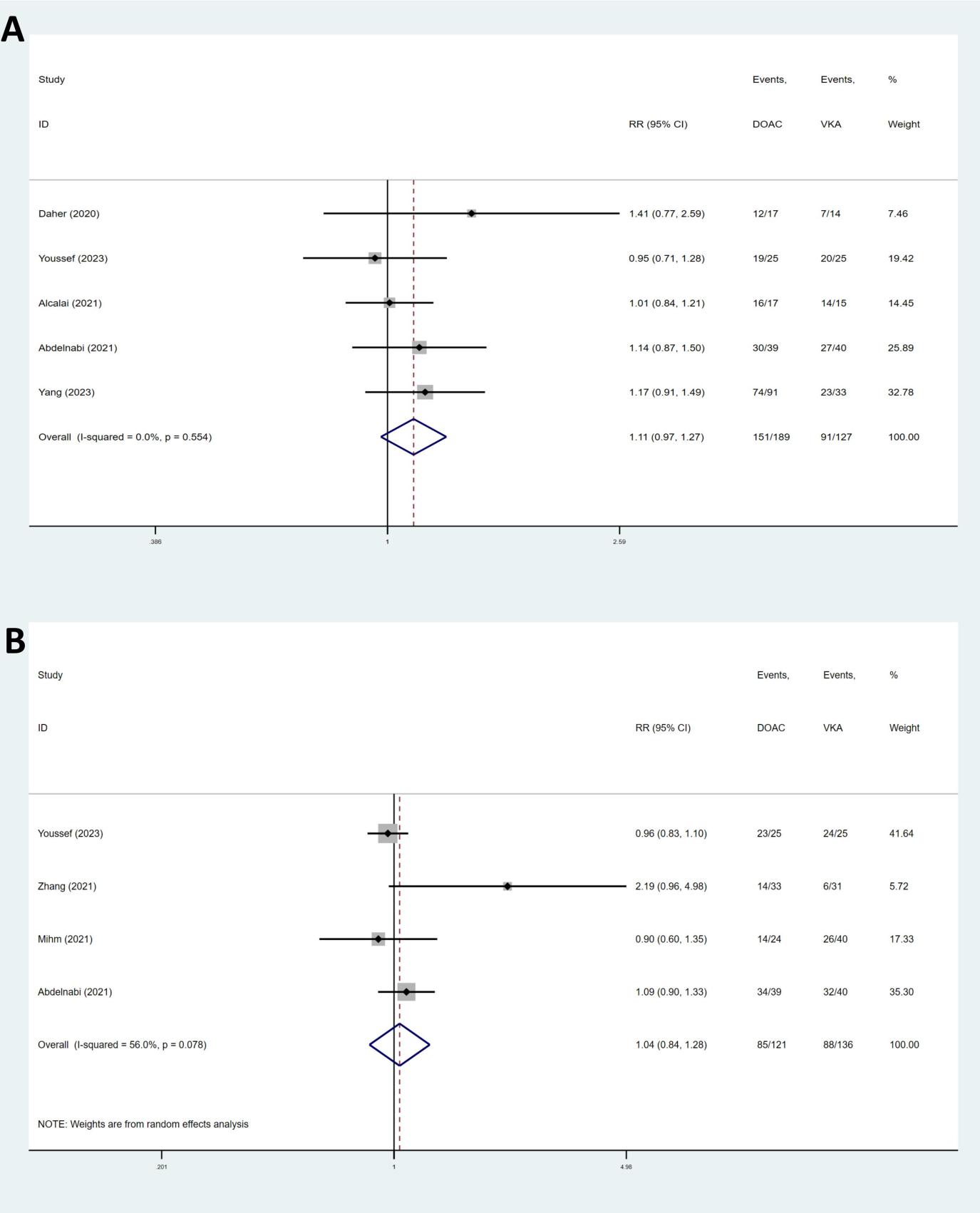
**

***Supplementary Figure 3.*** Forest plot to compare apixaban with VKAs in outcomes including (A) LVT resolution. (B) stoke/systemic embolism. (C) any bleeding.

**
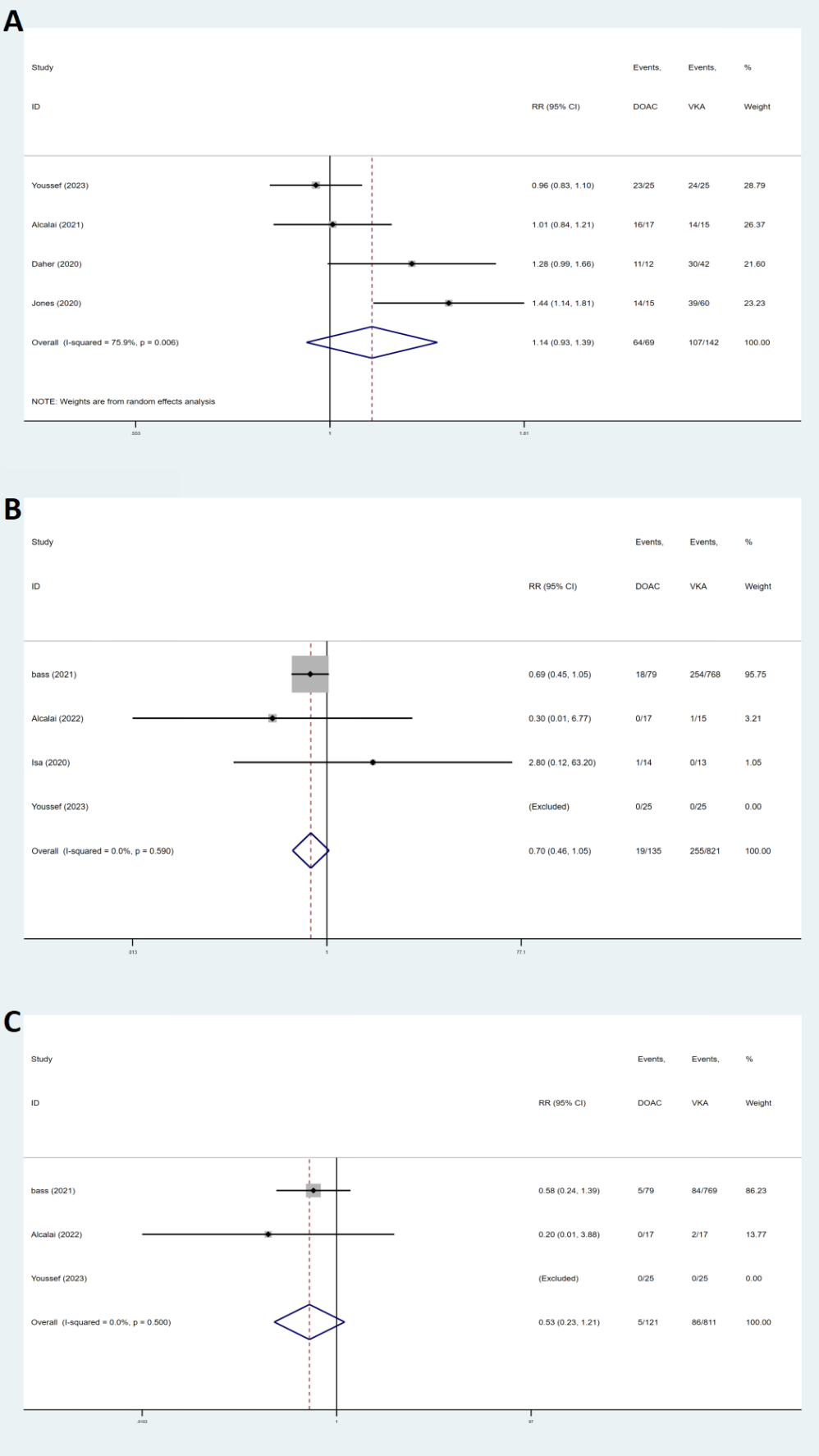
**

***Supplementary Figure 4.*** Forest plot to compare rivaroxaban with VKAs in outcomes including (A) LVT resolution. (B) stroke. (C) stroke/systemic embolism. (D) major bleeding. (E) any bleeding.

**
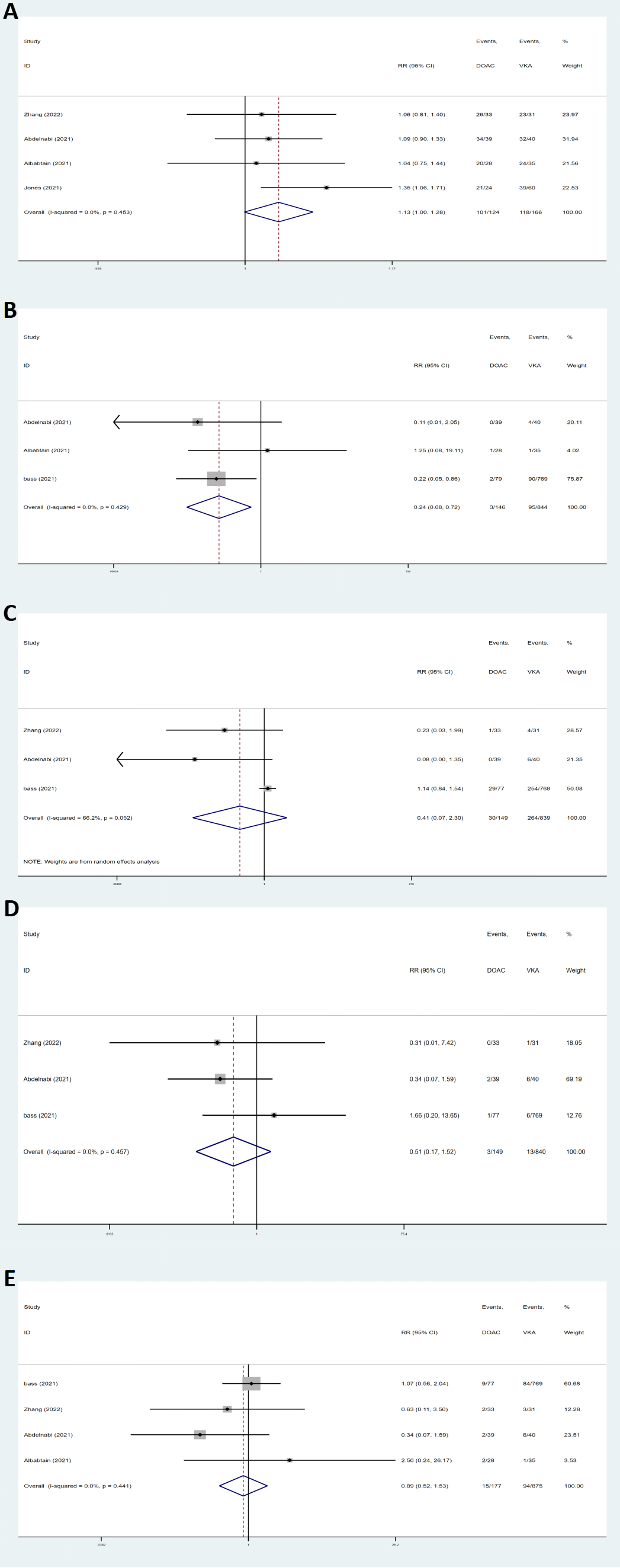
**

***Supplementary Figure 5.*** The funnel plots based on the outcomes. (A) funnel plot for LVT resolution. (B) funnel plot for stroke. (C) funnel plot for systemic embolism. (D) funnel plot for stroke/systemic embolism. (E) funnel plot for all-cause mortality. (F) funnel plot for any bleeding. (G) funnel plot for major bleeding events.

**
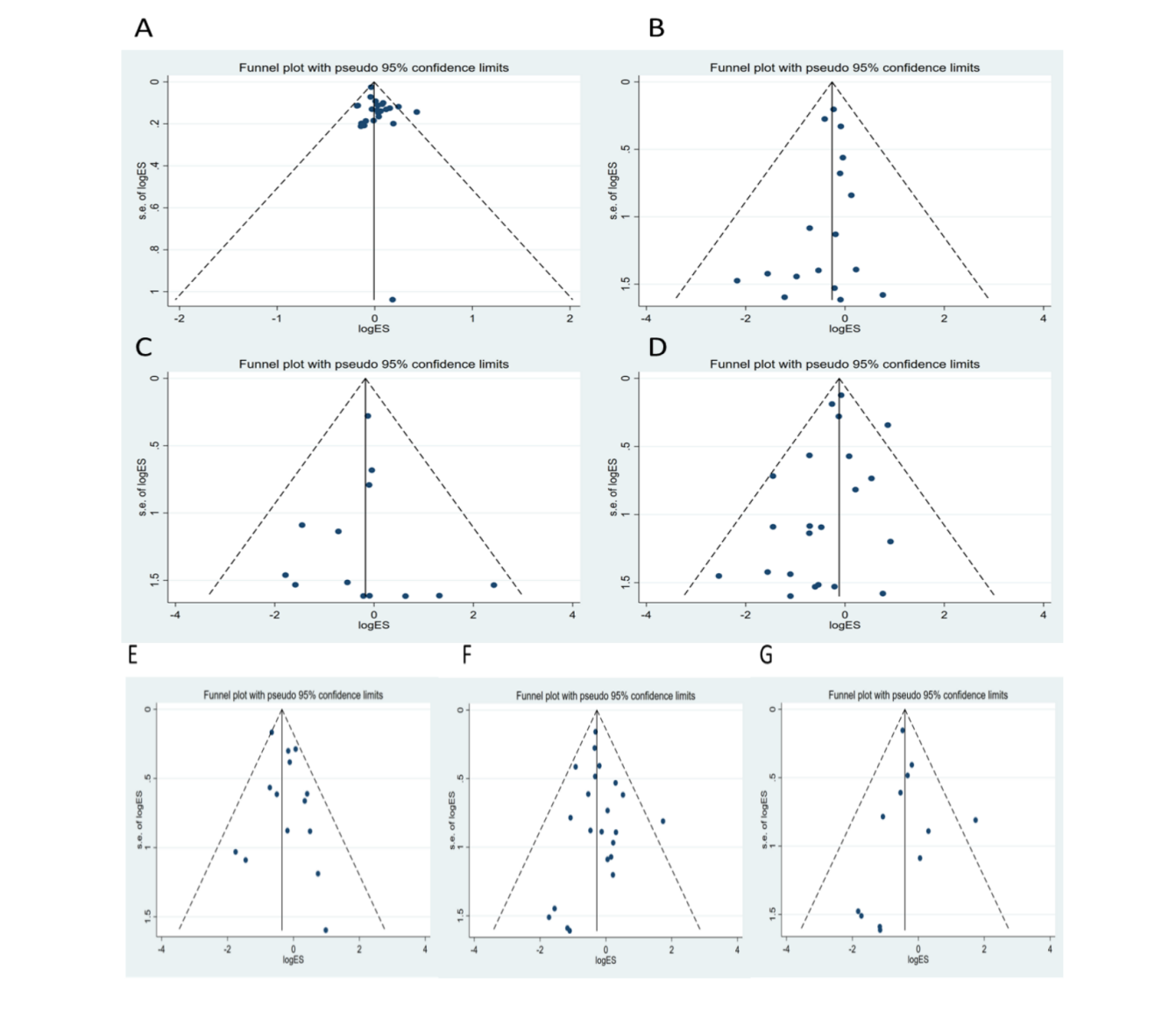
**

***Supplementary Figure 6.*** Results of sensitivity analyses. (A) LVT resolution. (B) ischemic stroke. (C) systemic embolism. (D) stroke/systemic embolism. (E) any bleeding. (F) major bleeding. (G) intracranial hemorrhage. (H) all-cause mortality. (I) adverse cardiovascular events.


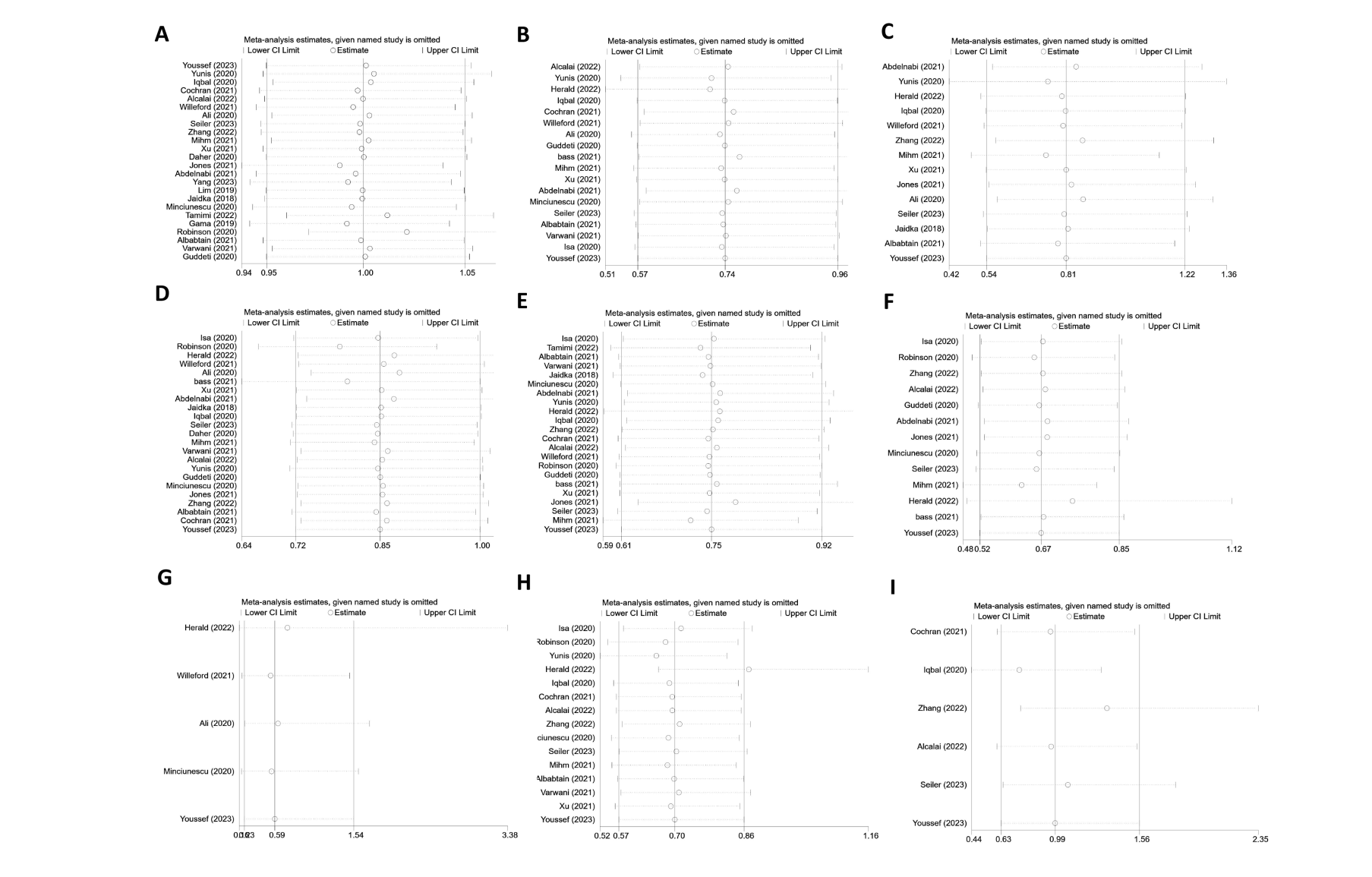

Supplement: Supplementary file 1 — Additional file 1: Supplementary Table 1. The detailed search strategy of databases. Supplementary Table 2. Quality assessment of included observational studies using the Newcastle-Ottawa Scale. Supplementary Figure 1. Quality assessment of the included randomized controlled trials. Supplementary Figure 2. Forest plot comparing the efficacy of DOACs and VKA in the treatment of LVT. (A) after 3 months of treatment. (B) after 6 months of treatment. Supplementary Figure 3. Forest plot to compare apixaban with VKAs in outcomes including (A) LVT resolution, (B) stoke/systemic embolism, (C) any bleeding. Supplementary Figure 4. Forest plot to compare rivaroxaban with VKAs in outcomes including (A) LVT resolution. (B) stroke. (C) stroke/systemic embolism. (D) major bleeding. (E) any bleeding. Supplementary Figure 5. The funnel plots based on the outcomes. (A) funnel plot for LVT resolution. (B) funnel plot for stroke. (C) funnel plot for systemic embolism. (D) funnel plot for stroke/systemic embolism. (E) funnel plot for all-cause mortality. (F) funnel plot for any bleeding. (G)funnel plot for major bleeding events. Supplementary Figure 6. Results of sensitivity analyses. (A) LVT resolution. (B) ischemic stroke. (C) systemic embolism. (D) stroke/systemic embolism. (E) any bleeding. (F) major bleeding. (G) intracranial hemorrhage. (H) all-cause mortality. (I) adverse cardiovascular events. [file 12959_2024_585_MOESM1_ESM.docx]
